# Quantifying Radiation Exposure Across Cardiac Catheterization Procedures

**DOI:** 10.3390/diagnostics16111636

**Published:** 2026-05-27

**Authors:** Md Fakhrul Islam Khaled, Mohammad Walidur Rahman, S M Ear E Mahabub, Sharmin Ahmed, Mohammad Moynul Hoque Munna, Md Mazharul Islam, Mahmud Hasan Mostofa Kamal, S M Mustafa Zaman, Sajal Krishna Banerjee, Mohammad Rayhan Masum Mandal

**Affiliations:** 1 Department of Cardiology, Bangladesh Medical University, Dhaka 1000, Bangladesh; smemahbub@gmail.com (S.M.E.E.M.); moynulmunna@gmail.com (M.M.H.M.); drsajalk2003@bsmmu.edu.bd (S.K.B.); rayhanmandal123@gmail.com (M.R.M.M.); 2 Department of Cardiology, Bangladesh Specialized Hospital, Dhaka 1207, Bangladesh; sharmin.disha@gmail.com; 3 Department of Animal Resources, Ministry of Municipality, Doha, Qatar; walidbdvet@gmail.com; 4 Department of Radiology and Imaging, Bangladesh Medical University, Dhaka 1000, Bangladesh; mkamal@bsmmu.edu.bd

**Keywords:** cardiac catheterization, radiation exposure, fluoroscopy, interventional cardiology, occupational radiation, ALARA, coronary angiography, percutaneous coronary intervention

## Abstract

**Background:** Coronary angiography and percutaneous coronary intervention are essential procedures for managing coronary artery disease but expose patients and healthcare personnel to ionizing radiation. Radiation exposure varies with procedural complexity and vascular access route, and repeated occupational exposure in catheterization laboratories (cath-labs) has been linked to serious health hazards among operators. **Objectives:** Given the high procedural volume and limited routine monitoring at Bangladesh Medical University (BMU), Bangladesh, this study aimed to quantify operator radiation exposure and identify factors influencing radiation dose. **Methods:** This analytical cross-sectional study was conducted from November 2023 to April 2024 in two cardiac cath-labs at BMU using two Siemens Axiom Artis angiography systems. Patient, operator, and procedural data, including demographics, operator experience, procedure type, vascular access route, fluoroscopic view, procedure duration, and radiation exposure, were collected from randomly selected routine procedures. Operator radiation exposure was measured at four working positions using a dosimeter, and cumulative annual exposure was estimated based on procedural workload. Descriptive, univariable, and multivariable analyses were performed to identify factors associated with radiation exposure. **Results:** The study analyzed 776 procedures, in which most patients were aged 46–65 years and male. The majority of procedures were coronary angiography performed via the femoral route. On average, each procedure required 14.28 fluoroscopic views and lasted 8.16 min, with a mean radiation exposure of 1034.5 mGy. Procedural complexity, radial access, and stent use were associated with higher fluoroscopic views, longer procedure time, and increased radiation exposure. Multivariable analysis showed that procedure type and stent number are primarily determinants for the number of fluoroscopic views, while procedure time was mainly driven by imaging demand and vascular access route. Radiation exposure was strongly associated with both procedure time and the number of views, and was higher among male and older patients but slightly lower with femoral access and among older operators. Direct measurements showed higher radiation levels near the operator’s X-ray beam, while estimated annual operator exposure remained low inside the lead apron (0.03–0.05 mSv) compared with outside the lead apron (0.6–1.1 mSv), which is within the limit of internationally accepted cumulative absorbed radiation. **Conclusions:** This study provides the first comprehensive evaluation of operator radiation exposure in a cardiac cath-lab in the country and the wider region. Procedural characteristics, particularly fluoroscopy use and procedural complexity, were the primary determinants of radiation exposure, while effective shielding and increased distance substantially reduced operator dose. These findings highlight the importance of imaging optimization and consistent implementation of the ALARA principle through structured radiation safety training and careful procedural planning to further minimize occupational exposure.

## 1. Introduction

Coronary angiography (CAG) and percutaneous coronary intervention (PCI) are cornerstone diagnostic and therapeutic procedures in the management of coronary artery disease. Both procedures rely on fluoroscopic imaging, which inevitably exposes patients and healthcare personnel to ionizing radiation. Despite substantial technological advancements in imaging systems and protective equipment, radiation safety remains a major concern in interventional cardiology because of the cumulative biological effects associated with repeated exposure.

Radiation exposure during CAG and PCI varies widely and is influenced by procedural duration, lesion complexity, patient body habitus, and technical factors. Recent studies have demonstrated marked variability in fluoroscopy time and dose–area product, highlighting the need for systematic radiation-optimization strategies in contemporary catheterization laboratories (cath-labs) [1]. Vascular access choice may also influence radiation exposure. Although radial access is now widely preferred for its clinical advantages, operator radiation exposure may be higher in certain radial procedures due to additional fluoroscopic angulations and closer proximity to the X-ray source [2]. Interventional operators receive an average effective dose of approximately 2.3 microSieverts (μSv) per PCI procedure using radial access and 1.2 μSv using femoral access [3]. Occupational exposure is therefore of particular concern for interventional cardiologists and cath-lab staff, who may accumulate substantial lifetime radiation doses despite routine use of lead shielding. Epidemiological studies have reported increased risks of radiation-induced cataracts and other stochastic effects among cath-lab personnel [4], and cumulative occupational radiation exposure has been associated with an elevated risk of malignancies, including lung, thyroid, and liver cancers [5]. Reflecting this growing concern, recent European Society of Cardiology guidelines emphasize the importance of continuous dose monitoring, appropriate protective equipment, and structured operator training [6,7]. Recent reports indicate that typical annual effective doses for interventional cardiologists now range between 1 and 5 mSv, even for high-volume operators [8,9]. The International Commission on Radiological Protection recommends maintaining occupational radiation exposure within 20 mSv per year, averaged over a five-year period, to minimize long-term stochastic risks [10].

Bangladesh Medical University (BMU) is a high-volume cardiac catheterization center, performing approximately 4400 procedures annually. Despite this substantial procedural workload, systematic measurement and monitoring of operator radiation exposure is not routinely implemented, and awareness of the cumulative occupational dose remains limited. Given the potential long-term health risks associated with chronic radiation exposure and the need for evidence-based safety benchmarks, evaluating real-world radiation exposure in this setting is critically important. Therefore, the present study aimed to quantify operator radiation exposure and to identify patient, operator, and procedural factors associated with increased radiation dose in this high-volume cardiac cath-lab. These findings are expected to inform targeted radiation safety interventions, reduce occupational risk, and align local practices with contemporary international standards.

## 2. Materials and Methods

### 2.1. Study Setting

This analytical cross-sectional study was conducted over a six-month period from November 2023 to April 2024 in the two cath-labs of BMU. The cath-labs routinely perform a wide range of procedures, including CAG, PCI, peripheral angiography, temporary and permanent pacemaker implantation, and right heart catheterization.

All procedures were performed using Siemens Axiom Artis angiography systems (model: Artis Q Floor; Siemens Healthineers, Forchheim, Germany) installed in both cath-labs. During every procedure, fluoroscopic imaging and recording were performed at a frame rate of 15 frames per second. Routine maintenance and quality assurance of the system were conducted twice annually in accordance with the manufacturer’s recommended protocols, including comprehensive electrical, mechanical, and radiation safety checks, as well as calibration of the X-ray tube and detector. For radiation protection, operators used a ceiling-mounted radiation protection shield and wore 0.5 mm lead-equivalent aprons with thyroid guards.

### 2.2. Patient and Operator Data

Cases for this study were randomly selected during routine cath-lab activities, with six patients included each working day. Randomization was achieved by enrolling every alternate patient among the first twelve scheduled procedures of the day. Informed consent was obtained from both patients and operators prior to data collection.

Patient age was categorized into four groups: ≤45 years, 46–55 years, 56–65 years, and >65 years. Operator age was categorized as ≤45 years, 46–55 years, and >55 years, while operator experience was grouped into <5 years, 5–10 years, and >10 years. Patient and operator sex were recorded as male or female.

### 2.3. Procedural Data

Procedural parameters included the type of intervention (CAG, PCI, combined CAG + PCI, and other procedures such as permanent pacemaker implantation and right heart catheterization), vascular access site (femoral or radial), and stent placement (single, multiple, or none).

Numerical procedural data included the number of fluoroscopic views or cine runs (View), defined as the number of recorded imaging sequences during contrast injection; fluoroscopy time (Time), expressed in minutes and representing the total duration of X-ray exposure during the procedure; and radiation exposure (Radiation), defined as cumulative air kerma measured in milligray (mGy) per procedure, automatically recorded by the angiography system at the reference point. All data were directly exported from the angiography system and incorporated into the study dataset.

### 2.4. Operator Radiation Exposure

Operator radiation exposure was estimated using a Geiger–Müller-based radiation survey meter (Radiation Alert^®^ Inspector USB, S.E. International Inc., Oak Ridge, TN, USA), which provides real-time measurements of ambient dose equivalent in microSieverts per hour (µSv/h). Measurements were taken at four predefined locations representing typical operator working positions: (i) above the X-ray beam projector at the operator site, (ii) outside the lead apron, (iii) inside the lead apron, and (iv) at the nursing/foot end of the patient. Each measurement was repeated three times, and the mean value was calculated.

For occupational risk assessment, cumulative annual operator radiation exposure was estimated for the three operators with the highest procedural volumes by combining mean exposure rate (µSv/h) with total estimated exposure time per year. Annual procedural volumes were proportionally extrapolated from each operator’s procedural share in the study sample using the institutional annual workload of approximately 4400 procedures. These measurements were location-based and used to provide an approximate estimation of occupational radiation exposure rather than individual dosimetric assessment.

### 2.5. Data Analysis

Descriptive statistics were calculated for all study variables. Categorical variables, such as patient age and sex, operator age, sex, and experience, procedure type, stent placement, and vascular access route, were summarized as frequencies (n), percentages (%), and 95% confidence intervals (CIs). Differences in categorical distributions were evaluated using the chi-square test. Continuous variables, including View, Time, and Radiation, were summarized using the mean, standard deviation (SD), median, interquartile range (IQR), and 95% CI of the mean.

Normality of continuous variables was assessed using the Shapiro–Wilk test and visually inspected with quantile–quantile (Q–Q) plots, both overall and stratified by relevant categories.

To examine the distribution of cases across categorical variables, cross-tabulations were generated to summarize patient, operator, and procedural characteristics (n and %). Associations were assessed using the chi-square test of independence.

Univariate analyses were conducted to assess differences in continuous outcomes across categorical predictors. The Kruskal–Wallis test was applied for categorical variables with three or more groups, while the Wilcoxon rank-sum test was used for variables with two categories. Boxplots were generated to visualize group-wise distributions, with corresponding *p*-values displayed to facilitate interpretation.

Multivariable regression analyses were conducted to examine factors associated with Views, Time, and Radiation. Overdispersion in View was assessed using the dispersion parameter, and a dispersion parameter greater than 2 (two) was considered indicative of overdispersion. Accordingly, negative binomial regression was applied for View. Time and Radiation, being continuous, strictly positive, and right-skewed variables, were modeled using gamma regression with a log link. To account for logical dependencies among the outcomes, View was included as a covariate in the Time model, and both View and Time were included as covariates in the Radiation model.

Model results are presented as exponentiated coefficients (exp β) with corresponding 95% CIs and *p*-values. For the negative binomial model (View), exp(β) represents incidence rate ratios (IRRs). For the gamma regression models (Time and Radiation), exp(β) represents multiplicative effects, interpreted as percentage changes in the outcome.

Model performance was evaluated using deviance and Akaike Information Criterion (AIC). Residual diagnostics, including residual-versus-fitted and Q–Q plots, were used to assess model assumptions. Multicollinearity among predictors was assessed using variance inflation factors (VIF), with values below 5 considered indicative of acceptable collinearity. In addition, log-transformed linear regression models for Time and Radiation were also fitted, and consistency of results was assessed as a sensitivity analysis. A two-sided *p*-value < 0.05 was considered statistically significant, while *p*-values < 0.01 were considered highly significant.

## 3. Results

### 3.1. Descriptive Statistics

A total of 900 cases were initially recorded and transferred to MS Excel for analysis. After data cleaning and verification, 124 records were excluded due to duplicate entries and incomplete or erroneous data fields. The final analysis included 776 complete cases (Table 1). The majority of patients were aged 46–65 years (*n* = 310, 39.95%, 95% CI: 36.50–43.50) and were male (*n* = 612, 78.87%, 95% CI: 75.79–81.65). Most of the procedures were performed by male operators (*n* = 745, 96.01%, 95% CI: 94.31–97.23), operators aged 46–55 years (*n* = 391, 50.39%, 95% CI: 46.81–53.96) and those with the highest level of experience (≥10 years; *n* = 420, 54.12%, 95% CI: 50.54–57.66).

Regarding procedural characteristics, CAG was the most frequently performed procedure (*n =* 387, 49.87%, 95% CI: 46.30–53.45), with the femoral route being the predominant access site (*n =* 597, 76.93%, 95% CI: 73.77–79.82). Among patients undergoing stent placement, the majority received a single stent (*n =* 197, 25.38, 95% CI: 22.39–28.63).

The average number of Views per patient was 14.28 ± 10.25 (95% CI: 13.55–15.00), with an average fluoroscopic Time of 8.16 ± 7.53 min (95% CI: 7.63–8.69). Mean Radiation was 1034.5 ± 988.84 mGy (95% CI: 964.78–1104.15) per procedure (Table 2).

### 3.2. Normality Assessment

The Shapiro–Wilk test indicated that the distributions of View, Time, and Radiation deviated significantly from normality (*p* < 0.01). Stratification by categorical predictors yielded similar results, confirming non-normality across groups. Q–Q plots showed systematic upward curvature in the tails, consistent with right-skewed distributions (Supplementary Figure S1).

### 3.3. Case Distribution

Cross-tabulation analyses of the cases showed that case allocation was associated with interrelated patient, procedural, and operator characteristics (Supplementary Table S1). Procedure type varied by patient age (*p* = 0.011) and sex (*p* < 0.001). Younger patients (≤45 years) more frequently underwent diagnostic CAG (60.9%), whereas middle-aged patients (46–65 years) had a higher proportion of combined CAG + PCI procedures (38.4–40.8%). Female patients predominantly underwent CAG (64.0%), while male patients more frequently underwent PCI (12.9%) and combined CAG + PCI (38.1%). Stent uses also varied by patient age (*p* = 0.036) and sex (*p* < 0.001), with higher proportions of single and multiple stent placement observed among middle-aged patients and males.

Operator age (*p* < 0.001) and experience (*p* < 0.001) were found to influence procedure type, with older and more experienced operators more frequently performing PCI and combined CAG + PCI procedures. Stent use showed a similar pattern (*p* < 0.001), with higher proportions of single and multiple stent placement observed among operators with greater experience. Access route showed limited association with patient and operator characteristics; however, it varied significantly by procedure type (*p* = 0.010), with femoral access more commonly used in PCI procedures.

### 3.4. Univariate Analysis

#### 3.4.1. Fluoroscopic View

**Figure 1:**
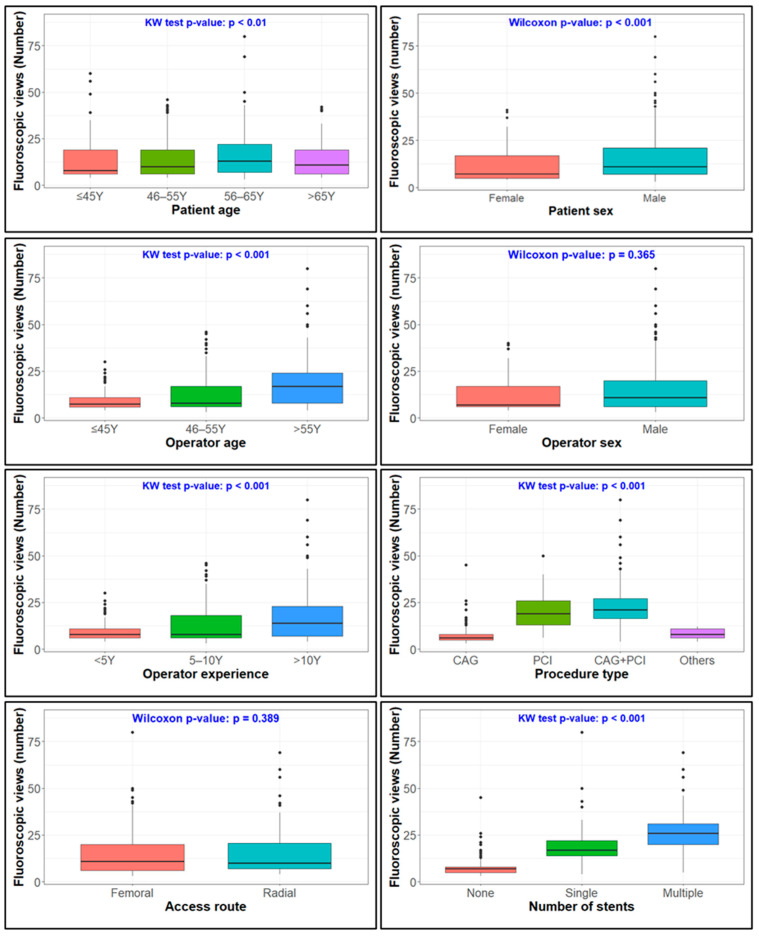
Univariate assessment of view with patient, operator, and procedural predictors.

The number of Views per procedure was significantly associated with patient age and sex, operator age and experience, procedure type and route, and the number of stents (Figure 1). Male patients required more Views (14.97 ± 10.49) than female patients (11.68 ± 8.87). Among patient age groups, those aged 56–65 years required the highest number of Views (16.00 ± 11.52), followed by 46–55 years (13.89 ± 9.40), >65 years (13.33 ± 8.95), and ≤45 years (13.09 ± 10.58).

Operators aged >55 years required more Views (17.83 ± 11.75) compared to those aged 46–55 years (12.38 ± 8.68) and ≤45 years (9.57 ± 5.83). Similarly, operators with >10 years of experience required more Views (16.35 ± 11.19) than those with 5–10 years (12.53 ± 8.95) or <5 years of experience (9.52 ± 5.66).

Regarding procedure type, CAG (7.08 ± 3.31) and other procedures (8.57 ± 2.68) required fewer Views compared to PCI alone (20.49 ± 8.86) and combined CAG + PCI (22.71 ± 9.70). Procedures performed via the radial route required slightly more Views (14.80 ± 11.11) than those performed via the femoral route (14.12 ± 9.98). Procedures involving multiple stents required the highest number of Views (26.63 ± 9.42), followed by single stent placement (18.43 ± 7.88).

#### 3.4.2. Fluoroscopy Time

**Figure 2:**
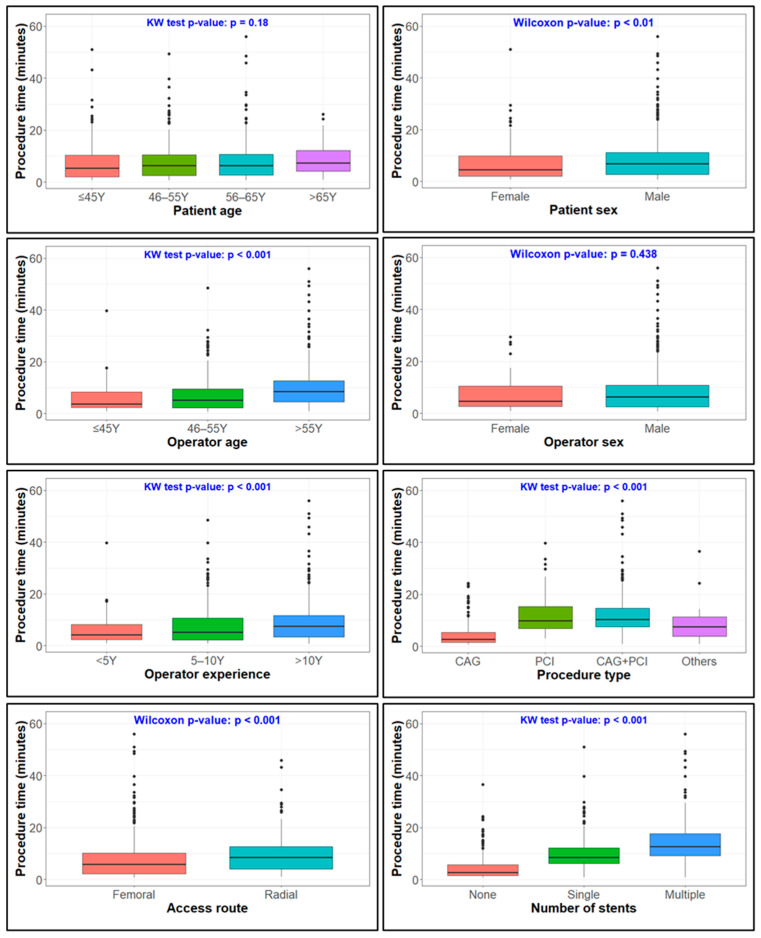
Univariate assessment of time with patient, operator, and procedural predictors.

Average Time per procedure was significantly influenced (*p* < 0.01) by patient sex, operator experience, procedure type and route, and the number of stents (Figure 2). Male patients required longer Time (8.49 ± 7.64 min) compared to female patients (6.93 ± 7.02 min).

Operators with >10 years of experience required the longest Time (8.91 ± 7.79 min), followed by those with 5–10 years (7.72 ± 7.47 min) and <5 years of experience (5.84 ± 5.66 min).

Procedural type also affected Time: combined CAG + PCI required the longest duration (12.52 ± 8.18 min), followed by PCI alone (11.94 ± 7.74 min), other procedures (8.95 ± 8.34 min), and CAG alone (4.11 ± 3.87 min). Procedures performed via the radial route required longer Time (9.98 ± 7.74 min) compared to the femoral route (7.62 ± 7.39 min). Multiple stent procedures required significantly more Time (14.92 ± 8.94 min) than single stent procedures (10.25 ± 6.54 min).

#### 3.4.3. Radiation Exposure

**Figure 3:**
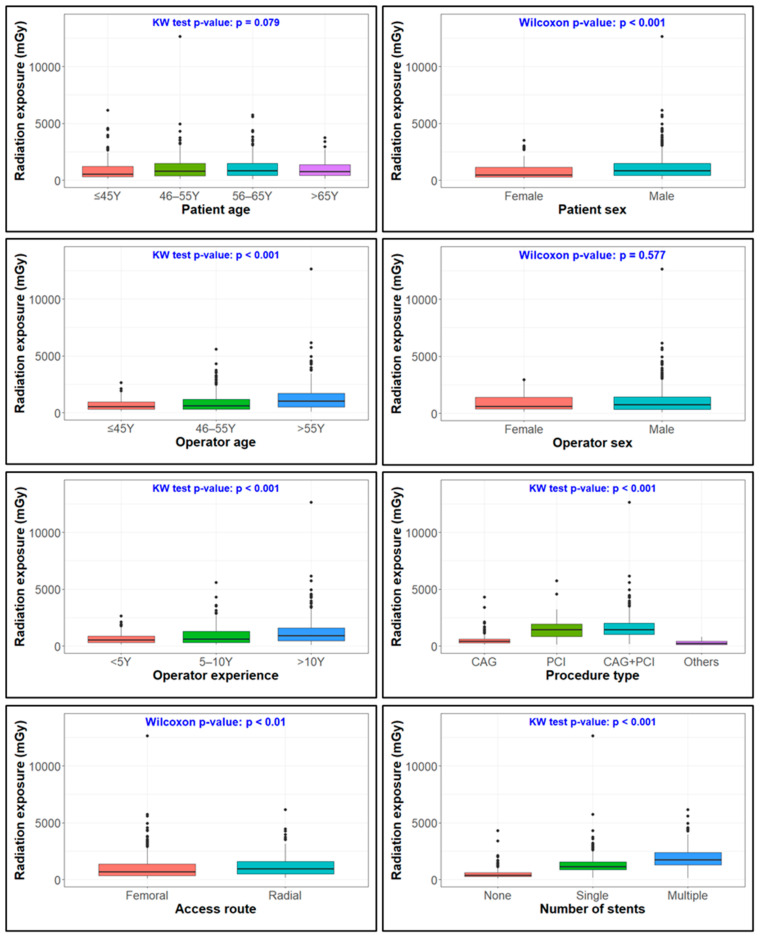
Univariate assessment of radiation with patient, operator, and procedural predictors.

Radiation was significantly associated with patient sex, operator age and experience, procedure type and route, and number of stents (Figure 3). Operators were exposed to significantly higher Radiation (*p* < 0.01) when performing procedures on male patients (1104.70 ± 1037.93 mGy) compared to female patients (772.36 ± 723.34 mGy).

Operator age was a significant factor (*p* < 0.01), with those aged >55 years experiencing the highest Radiation (1306.88 ± 1208.92 mGy), followed by 46–55 years (880.92 ± 794.36 mGy) and ≤45 years (716.85 ± 538.47 mGy). Similarly, operators with >10 years of experience received higher Radiation (1178.02 ± 1117.79 mGy) than those with 5–10 years (918.41 ± 832.46 mGy) and <5 years of experience (686.96 ± 531.06 mGy) (*p* < 0.01).

Procedural type strongly influenced Radiation: combined CAG + PCI generated the highest exposure (1686.96 ± 1144.78 mGy), followed by PCI alone (1495.07 ± 905.57 mGy), CAG alone (502.92 ± 406.04 mGy), and other procedures (283.54 ± 206.14 mGy). Procedures performed via the radial route resulted in higher Radiation (1187.72 ± 976.95 mGy) compared to the femoral route (988.51 ± 988.56 mGy). Finally, stent use was a key determinant: procedures involving multiple stents were associated with the highest Radiation (1932.63 ± 1014.19 mGy), followed by single stents (1385.81 ± 1100.46 mGy), and procedures without stents (495.43 ± 407.31 mGy).

### 3.5. Multivariate Analysis

#### 3.5.1. Fluoroscopic View

In the negative binomial regression model, Views were primarily influenced by procedural characteristics, with limited contribution from patient and operator factors (Table 3). Combined CAG + PCI procedures required significantly more Views compared to CAG alone (89% increase, 95% CI: 12–220, *p* = 0.016). Procedures involving multiple stents required 44% more Views than those with a single stent (95% CI: 34–54, *p* < 0.001).

#### 3.5.2. Fluoroscopy Time

In the gamma regression model for Time, Views were strongly associated with longer duration (4.9% increase per additional View, 95% CI: 4.1–5.8, *p* < 0.001) (Table 4). Femoral access was associated with shorter Time compared to radial access (28% reduction, 95% CI: 19–37, *p* < 0.001), whereas other procedures were associated with substantially longer duration compared to CAG (119% increase, 95% CI: 58–210, *p* < 0.001).

#### 3.5.3. Radiation Exposure

In the gamma regression model for Radiation, both Time and Views were strongly associated with radiation dose (Table 5). Each additional View increased radiation by approximately 4% (95% CI: 2.9–4.5, *p* < 0.001), while each additional minute of Time increased Radiation by approximately 4.0% (95% CI: 3.2–4.9, *p* < 0.001).

Among patient-related factors, patients aged 46–55 years also showed increased exposure (15% increase, 95% CI: 3–27, *p* = 0.010) and male patients had higher Radiation (21% increase, 95% CI: 11–33, *p* < 0.001). Operators aged 46–55 years had lower Radiation compared with those aged ≤45 years (36% reduction, 95% CI: 1–58, *p* = 0.043).

Femoral access was associated with a modest reduction in Radiation (10% lower, 95% CI: 1–17, *p* = 0.027), and other procedures were associated with substantially lower Radiation compared to CAG (54% reduction, 95% CI: 41–63, *p* < 0.001). Procedures involving multiple stents were associated with a modest reduction in Radiation (14% lower, 95% CI: 4–24, *p* = 0.01).

#### 3.5.4. Model Performance and Sensitivity Analysis

Model performance evaluation indicated adequate fit for both the negative binomial and gamma regression models. The negative binomial model for Views appropriately accounted for overdispersion (dispersion parameter = 2.29). Deviance, Akaike Information Criterion (AIC), and residual diagnostics supported acceptable model performance. Residual-versus-fitted and Q–Q plots did not indicate major model violations. Multicollinearity among predictors was low, with all adjusted VIF values below 3.5.

Sensitivity analyses using log-transformed linear regression models for Time and Radiation yielded consistent results, supporting the robustness of the findings.

### 3.6. Operator Radiation Exposure

Measured operator radiation dose rates varied across locations in the cath-lab. The highest dose rate was recorded above the X-ray beam projector at the operator site (45.6 µSv/h), followed by outside the lead apron (8.4 µSv/h), inside the lead apron (0.42 µSv/h), and at the nursing/foot end of the patient (0.14 µSv/h).

Three interventional cardiologists performed the majority of procedures, contributing 166, 104, and 64 procedures, respectively, out of the 776 cases. Based on their proportional procedural distribution and an annual institutional workload, the estimated annual caseloads for these operators were approximately 972, 810, and 458 procedures, respectively. Using these estimated workloads, cumulative annual operator radiation exposure was calculated. Inside the lead apron, estimated annual exposure ranged from 23.0 to 51.2 µSv/year, whereas exposure outside the lead apron ranged from 459.6 to 1024.7 µSv/year (Table 6).

## 4. Discussion

This study provides insight into occupational radiation exposure among interventional cardiologists working in a high-volume cath-lab and identified patient-, operator-, and procedure-related factors associated with radiation burden. Understanding these determinants is essential for strengthening radiation safety practices in interventional cardiology, particularly in settings with increasing procedural complexity and workload. As one of the first studies from Bangladesh to address this issue comprehensively, the findings contribute region-specific evidence to the global literature on cath-lab radiation safety.

Cardiac catheterization is a technically demanding procedure. Most operators in this study were aged 45–55 years and had more than 10 years of experience, indicating that the BMU Cardiac Centre is largely staffed by experienced cardiologists. However, the marked underrepresentation of female operators mirrors global trends and highlights persistent structural and occupational barriers within interventional cardiology. Addressing concerns related to radiation exposure, career sustainability, and work–life balance may be essential for improving gender equity in this field [11].

Regarding procedural characteristics, CAG remained the most common procedure, while femoral access was more frequent than the increasingly radial-dominant pattern reported in many contemporary centers. However, the study found that the femoral route results in less Views, Time and Radiation, which may influence the operators to frequently select the femoral route. In addition, this likely reflects local operator preference, case selection, and institutional practice patterns. However, some confounding variables, such as patient body habitus, coronary lesion complexity, and detailed technical parameters (e.g., fluoroscopy settings and projection angles), were not included in the analysis, although the present findings reflect real-world practice in a major referral center and provide important baseline data for similar healthcare systems. Future studies should incorporate these variables to further refine understanding of factors influencing radiation exposure in the cath-lab.

Patient-related characteristics appear to influence operator radiation exposure indirectly rather than acting as independent determinants [1]. Factors such as male sex and older age are commonly associated with larger body habitus and more advanced coronary disease, which in turn lead to increased Views and scatter radiation and may necessitate prolonged or technically demanding interventions. However, once procedural factors are taken into account, the contribution of patient demographics diminishes, underscoring that radiation burden is largely procedure-driven. This interpretation aligns with previous reports suggesting that patient characteristics modify radiation exposure primarily by shaping procedural complexity rather than through intrinsic effects [1,12].

Operator-level characteristics reflect a balance between experience, procedural role, and technical efficiency. Univariate analyses suggested higher radiation exposure among older and more experienced operators. However, multivariable models revealed lower adjusted exposure among operators aged over 46 years. This shift likely reflects confounding by procedural allocation rather than a direct effect of operator experience.

In this study, junior operators predominantly performed diagnostic procedures, whereas more complex cases were handled by senior operators. As complex procedures require more Views and longer imaging time, the higher radiation observed in univariate analysis likely reflects case complexity. After adjustment for procedural factors, the underlying effect of operator expertise becomes evident, whereby experienced operators may achieve greater technical efficiency. Specifically, senior operators may minimize Time through more efficient catheter handling, optimized imaging angles, and greater adherence to Time-reduction techniques. Consequently, overall Radiation exposure may be reduced, consistent with previous reports among experienced operators [2].

Procedural factors remain the dominant contributors to radiation exposure in interventional cardiology [12]. In this study, Views, Time, and Radiation were strongly correlated. Multivariate analysis confirmed that procedure type, access route, and the number of implanted stents were the main determinants of radiation-related outcomes. PCI and combined CAG-PCI required substantially greater fluoroscopy use and produced higher radiation exposure than diagnostic angiography, reflecting the technical demands of guidewire navigation, device positioning, and stent deployment. Previous studies have similarly reported significantly higher radiation exposure during PCI [12]. Radial access was associated modestly with increased Time, likely attributable to anatomical variation and catheter manipulation challenges, consistent with the previous reports [6]. Furthermore, this center has recently adopted radial access and the learning curve of the radial access procedure takes more time [13]. Modest reduction in radiation with multi-stent deployment may be related to procedural allocation and technical expertise of senior operators. These findings suggest that radiation exposure results from interaction between procedural complexity and operator technique rather than procedure duration alone [2,12].

In this study, the highest exposure was recorded immediately above the X-ray beam, with substantially lower levels outside the lead apron, beneath the lead apron, and at the nursing or foot end of the patient table. These findings reflect the distribution of scattered radiation during X-ray-guided procedures and follow the inverse-square law and align with previous studies demonstrating that scatter radiation is greatest near the patient and the beam entrance site [2]. Operator-site radiation exposure was higher than values commonly reported in contemporary catheterization practice. This difference likely reflects continuous fluoroscopy during measurement and dosimeter placement at peak scatter locations. Nevertheless, the relative positional gradient observed remains clinically important and supports previous observations [1,14,15].

The marked attenuation of radiation beneath the lead apron (95% reduction) highlights the effectiveness of personal protective equipment, reinforcing the importance of lead aprons, thyroid shields, and ceiling-mounted or table-side shielding. Near-background radiation levels at the foot end emphasize distance as one of the most effective protection strategies, as even a 50–100 cm increase can markedly reduce scatter exposure. These findings have practical implications for workflow design and staff positioning in high-volume interventional settings [2].

Overall, these findings reinforce the fundamental principles of time, distance, and shielding and enhanced operator awareness as core components of radiation safety [16] and cornerstones of radiation safety in interventional imaging. In addition, the widespread implementation of modern fluoroscopic systems, featuring pulsed imaging, improved detector efficiency, and advanced image-processing algorithms, may have further contributed to Radiation reduction [17,18]. With increasing procedural volume and complexity observed worldwide, particularly in interventional cardiology, structured radiation safety training and continued reinforcement of protection protocols remain essential.

The three highest-volume operators in this study performed approximately half of all procedures yet demonstrated remarkably low estimated annual radiation doses at the operator site. When adjusted for protection beneath the lead apron, the effective annual doses were further reduced to only 22.98–51.23 µSv, values that are substantially lower than those reported in earlier studies and well below internationally recommended occupational dose limits [19]. This favorable exposure profile suggests that procedural volume alone does not necessarily translate into increased radiation risk and likely reflects a combination of heightened radiation safety awareness, strict adherence to the “As Low As Reasonably Achievable” (ALARA) principle, accumulated operator experience, and consistent use of optimized fluoroscopic techniques. Importantly, the estimated operator doses were also considerably lower than the average annual global background radiation exposure of approximately 2400 µSv [19], indicating an overall low-radiation working environment in this cath-lab.

This study has several limitations. The study was conducted in a single high-volume tertiary cardiac center using a single angiography system, which may limit generalizability to other institutions with different equipment and procedural protocols. A substantial proportion of procedures were performed using femoral access, whereas contemporary practice in many centers is increasingly radial-dominant. Operator radiation exposure in this study was estimated using location-based survey meter measurements at predefined positions rather than personal dosimetry (e.g., TLD/OSL). The reported values do not represent cumulative individual exposure and may not fully capture variability across procedures, operator positioning, and shielding practices. However, they provide useful insight into the relative magnitude of exposure in routine practice and may help contextualize operator radiation risk. Future studies incorporating repeated dosimeter measurements across multiple procedures, working environments, and analyzers; inclusion of additional clinical variables; and collaboration with medical physicists or radiation safety experts would further strengthen measurement accuracy and enhance the generalizability of the findings.

## 5. Conclusions

This study presents the first comprehensive evaluation of operator radiation exposure and its determinants in cardiac cath-labs in the country and the wider region. Procedural factors, particularly View, Time, and Radiation, procedure type, access route, and stent number emerged as the dominant drivers of radiation exposure, highlighting procedural optimization as the key determinant of occupational radiation burden. Despite high procedural volumes, the estimated annual operator doses were observed to be below recommended occupational limits. However, these findings should be interpreted with caution, as exposure was assessed during location-based measurements rather than personal dosimetry. The results nevertheless suggest that adherence to radiation protection practices, including shielding and optimizing fluoroscopic techniques, may contribute to maintaining lower exposure levels in routine practice. These findings emphasize the importance of continued radiation safety training, optimized procedural strategies, and consistent use of protective measures in modern cath-lab practice. Future multicenter studies with longitudinal and individual dosimetric monitoring, including eye and extremity dose assessment, are warranted to further refine exposure estimates and guide targeted radiation-reduction strategies.

## Figures and Tables

**Table 1:** Descriptive statistics about the variables.

Sl No	Variables	Categories	N	%, (95% CI)	*p* Value
1	Patient age	≤45 years	138	17.78 (15.19–20.70)	*p* < 0.01
		46–55 years	310	39.95 (36.50–43.50)	
		56–65 years	223	28.74 (25.60–32.09)	
		>65 years	105	13.53 (11.24–16.19)	
2	Patient sex	Female	164	21.13 (18.35–24.21)	*p* < 0.01
		Male	612	78.87 (75.79–81.65)	
3	Operator sex	Female	31	3.99 (2.77–5.67)	*p* < 0.01
		Male	745	96.01 (94.31–97.23)	
4	Operator age	≤45 years	76	9.79 (7.84–12.16)	*p* < 0.01
		46–55 years	391	50.39 (46.81–53.96)	
		>55 years	309	39.82 (36.37–43.37)	
5	Operator experience	<5 years	82	10.57 (8.54–13.00)	*p* < 0.01
		5–10 years	274	35.31 (31.96–38.80)	
		>10 years	420	54.12 (50.54–57.66)	
6	Treatment procedure	CAG	387	49.87 (46.30–53.45)	*p* < 0.01
		PCI	89	11.47 (9.36–13.97)	
		CAG + PCI	279	35.95 (32.59–39.46)	
		Others	21	2.71 (1.73–4.18)	
7	Treatment route	Radial	179	23.07 (20.18–26.23)	*p* < 0.01
		Femoral	597	76.93 (73.77–79.82)	
8	Stent	Single stent	197	25.38 (22.39–28.63)	*p* < 0.01
		Multi-stent	169	21.78 (18.96–24.88)	
		Not done	410	52.84 (49.25–56.39)	

CAG: coronary angiogram, PCI: percutaneous coronary intervention.

**Table 2:** Summary statistics of fluoroscopic view, fluoroscopic time, and radiation exposure dose per procedure.

Sl No	Variable (Unit)	Mean	Standard Deviation	95% CI	Median	Interquartile Range
1	Fluoroscopic view (n)	14.28	10.25	13.56–15.00	10.00	6.00–20.00
2	Fluoroscopic time (min)	8.16	7.53	7.63–8.69	6.30	2.60–10.90
3	Radiation exposure (mGy)	1034.50	988.84	964.78–1104.15	739.90	359.42–1432.00

**Table 3:** Multivariate regression analysis of fluoroscopic views (View) with patient, operator, and procedural predictors.

Variables (Reference)	Categories	Adjusted Effect (IRR)	95% CI	*p*-Value
(Intercept)	-	10.83	6.19–19.05	<0.001
Patient age (≤45 years)	46–55 years	0.96	0.89–1.04	0.365
	56–65 years	1.03	0.95–1.12	0.496
	>65 years	0.98	0.88–1.08	0.630
Patient sex (Female)	Male	1.07	1.00–1.15	0.052
Operator age (≤45 years)	46–55 years	1.16	0.80–1.65	0.427
	>55 years	1.26	0.87–1.80	0.218
Operator sex (Female)	Male	0.90	0.78–1.04	0.157
Operator Experience (<5 years)	5–10 years	0.81	0.58–1.16	0.242
	>10 years	0.82	0.58–1.17	0.257
Route (Radial)	Femoral	0.98	0.92–1.05	0.516
Procedure (CAG)	PCI	1.69	0.99–2.88	0.051
	CAG + PCI	1.89	1.12–3.20	0.016
	Others	1.16	0.95–1.40	0.137
Stent (Single)	None	0.73	0.43–1.22	0.227
	Multi	1.44	1.34–1.54	<0.001

**Table 4:** Multivariate regression analysis of procedure time (Time) with patient, operator, and procedural predictors.

Variables (Reference)	Categories	Adjusted Effect (Exp β)	95% CI	*p*-Value
(Intercept)	-	3.73	1.03–9.96	0.019
View		1.05	1.04–1.06	<0.001
Patient age (≤45 years)	46–55 years	1.05	0.91–1.21	0.491
	56–65 years	0.91	0.78–1.07	0.250
	>65 years	1.15	0.95–1.38	0.136
Patient sex (Female)	Male	1.05	0.80–1.31	0.724
Operator age (≤45 years)	46–55 years	0.88	0.47–1.64	0.679
	>55 years	1.00	0.53–1.90	0.990
Operator sex (Female)	Ma le	1.05	0.79–1.38	0.725
Operator experience (<5 years)	5–10 years	1.09	0.60–1.97	0.782
	>10 years	1.00	0.55–1.83	0.997
Route (Radial)	Femoral	0.72	0.63–0.81	<0.001
Procedure (CAG)	PCI	1.50	0.53–4.26	0.446
	CAG + PCI	1.36	0.49–3.77	0.563
	Others	2.19	1.57–3.06	<0.001
Stent (Single)	None	0.90	0.32–2.54	0.844
	Multi	0.95	0.81–1.12	0.508

**Table 5:** Multivariate regression analysis of radiation exposure (Radiation) with patient, operator, and procedural predictors.

Variables (Reference)	Categories	Adjusted Effect (Exp β)	95% CI	*p*-Value
(Intercept)	-	262.00	130.00–479.00	<0.001
View		1.04	1.03–1.04	<0.001
Time		1.04	1.03–1.05	<0.001
Patient age (≤45 years)	46–55 years	1.15	1.03–1.27	0.010
	56–65 years	1.03	0.92–1.15	0.587
	>65 years	1.14	1.00–1.30	0.056
Patient sex (Female)	Male	1.21	1.11–1.33	<0.001
Operator age (≤45 years)	46–55 years	0.64	0.42–1.01	0.043
	>55 years	0.66	0.44–1.06	0.071
Operator sex (Female)	Male	0.96	0.78–1.16	0.662
Operator experience (<5 years)	5–10 years	1.37	0.87–2.04	0.146
	>10 years	1.41	0.90–2.10	0.111
Route (Radial)	Femoral	0.90	0.83–0.99	0.027
Procedure (CAG)	PCI	1.61	0.83–3.66	0.201
	CAG + PCI	1.63	0.85–3.67	0.183
	Others	0.46	0.37–0.59	<0.001
Stent (Single)	None	1.12	0.58–2.52	0.764
	Multi	0.86	0.76–0.96	0.010

**Table 6:** Estimated cumulative annual occupational radiation exposure for the three operators with the highest procedural volumes *.

Operators	During Study Period		Estimated Annual			
	Caseload (n)	Fluoroscopy Time (Minutes)	Caseload (n)	Fluoroscopy Time (Hours)	Radiation Exposure Inside Lead Apron (µSv/Year)	Radiation Exposure Outside Lead Apron (µSv/Year)
1	166	1099.10	941	104	43.62	872.48
2	104	1290.80	590	122	51.23	1024.66
3	64	579.00	363	55	22.98	459.62

* Estimated using measured ambient dose rates of 0.42 µSv/h inside and 8.4 µSv/h outside the lead apron.

## Data Availability

We confirm that the data supporting the findings of this study will be shared upon reasonable request to the corresponding authors.

## Supplementary Materials

The following supporting information can be downloaded at: https://www.mdpi.com/article/10.3390/diagnostics16111636/s1.
