# Supplementary material for: Quantifying Radiation Exposure Across Cardiac Catheterization Procedures
Source: Diagnostics (Basel). 2026 May 27;16(11):1636. doi: 10.3390/diagnostics16111636 (PMC13257294; doi:10.3390/diagnostics16111636)

Supplementary figure S1:

1 Normality test: QQ plots

1.1 View

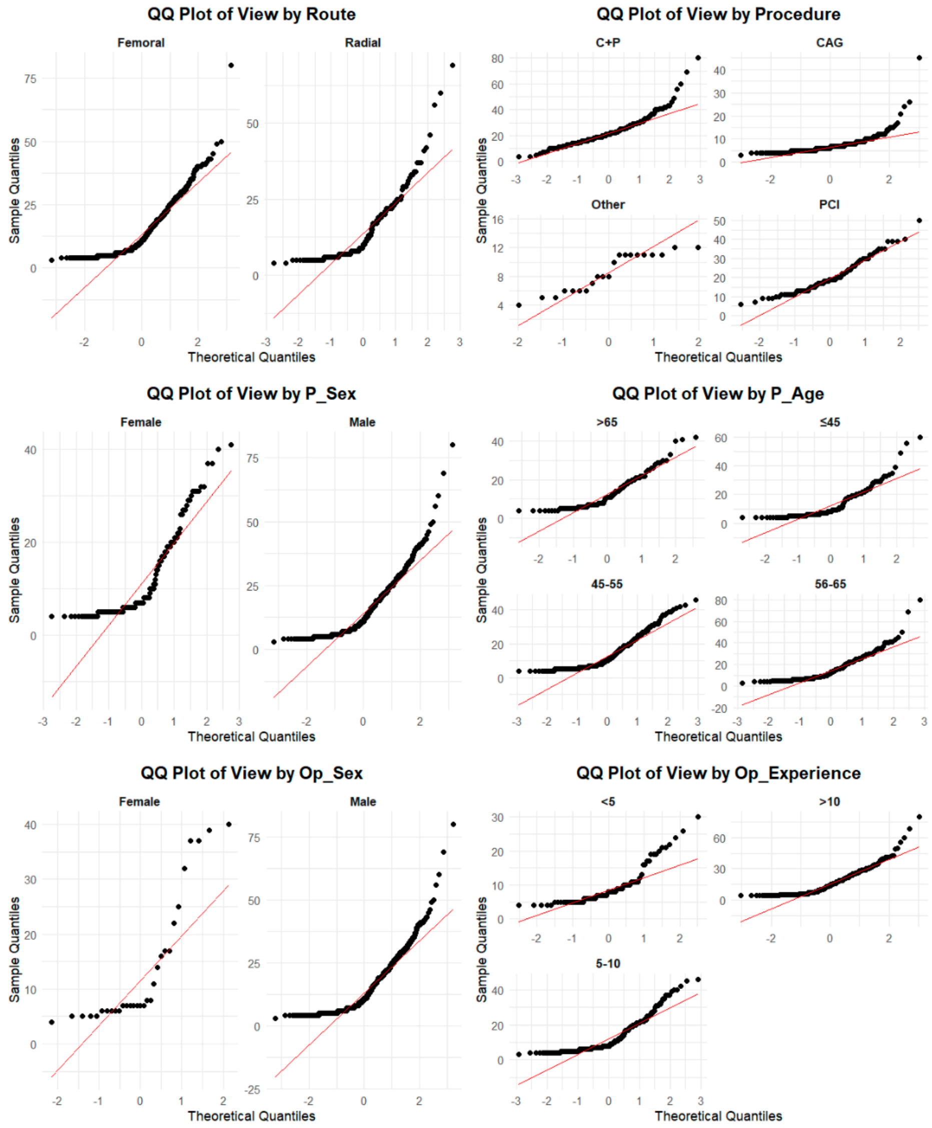

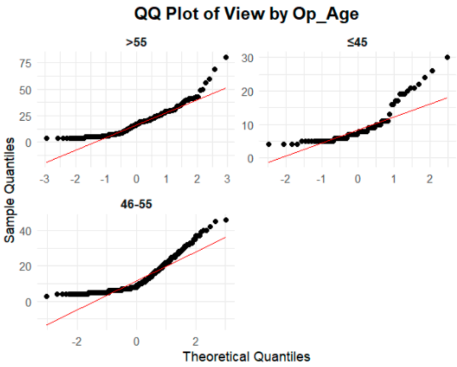
QQ Plot of View by Op_Age
>55
≤45
46-55
Sample Quantiles
Theoretical Quantiles

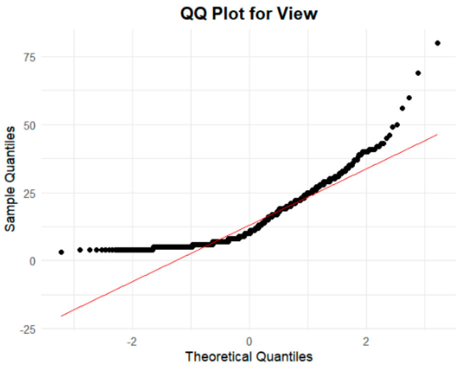
QQ Plot for View
Sample Quantiles
Theoretical Quantiles

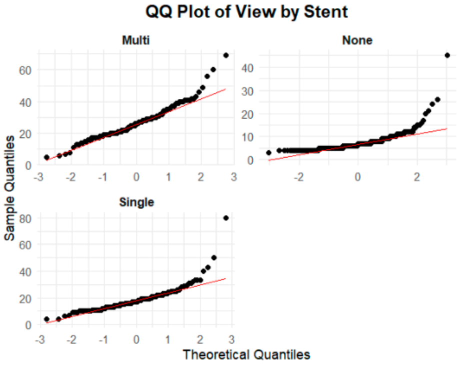
QQ Plot of View by Stent
Multi
None
Single
Sample Quantiles
Theoretical Quantiles

1.2 Time

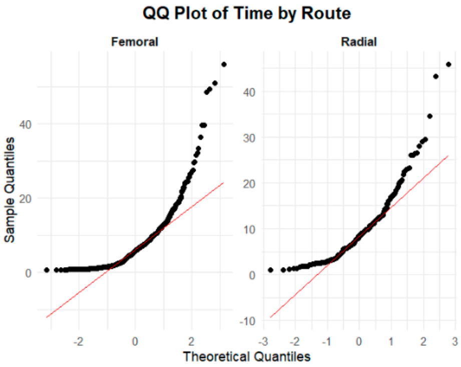
QQ Plot of Time by Route
Femoral
Radial
Sample Quantiles
Theoretical Quantiles

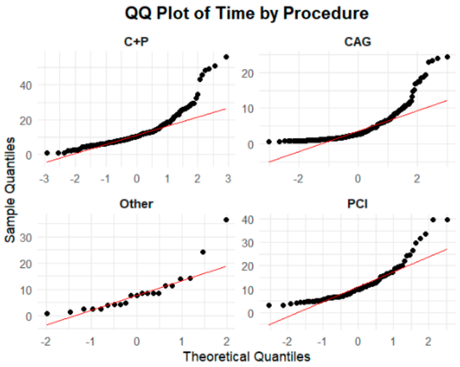
QQ Plot of Time by Procedure
C+P
CAG
Other
PCI
Sample Quantiles
Theoretical Quantiles

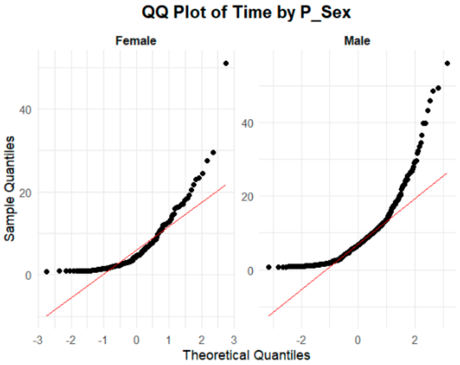
QQ Plot of Time by P_Sex
Female
Male
Sample Quantiles
Theoretical Quantiles

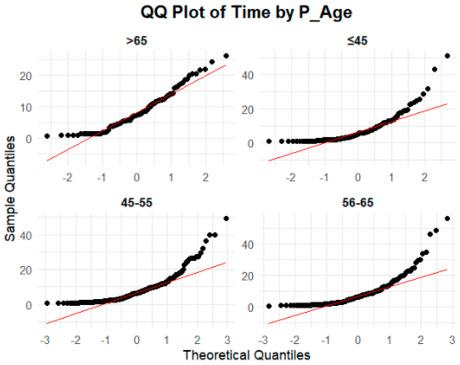
QQ Plot of Time by P_Age
>65
≤45
45-55
56-65
Sample Quantiles
Theoretical Quantiles

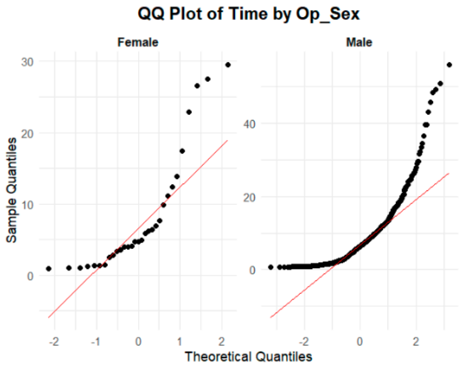
QQ Plot of Time by Op_Sex
Female
Male
Sample Quantiles
Theoretical Quantiles

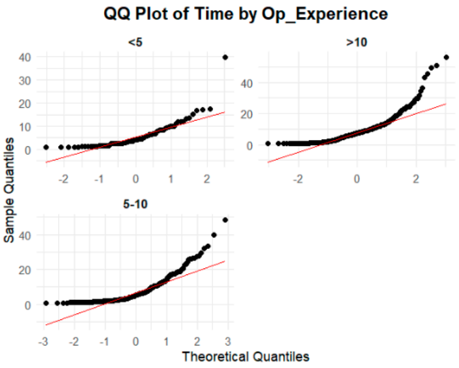
QQ Plot of Time by Op_Experience
<5
>10
5-10
Sample Quantiles
Theoretical Quantiles

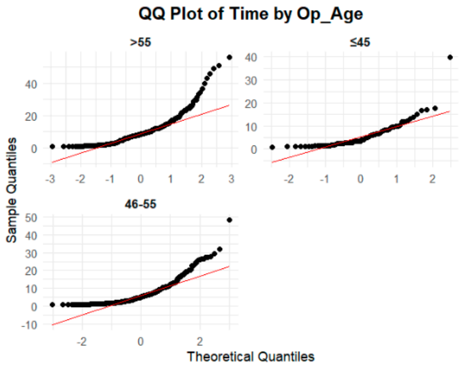
QQ Plot of Time by Op_Age
>55
≤45
46-55
Sample Quantiles
Theoretical Quantiles

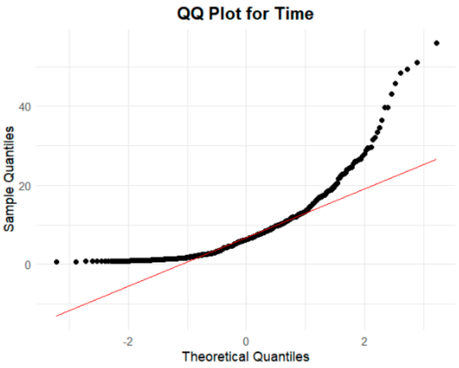
QQ Plot for Time
Sample Quantiles
Theoretical Quantiles

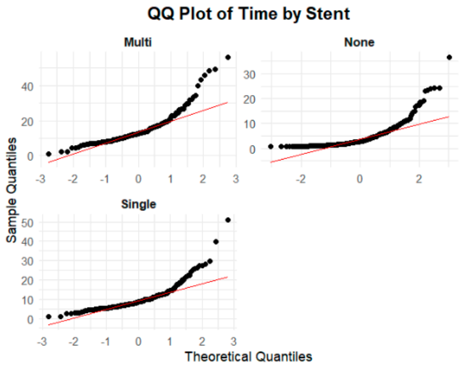
QQ Plot of Time by Stent
Multi
None
Single
Sample Quantiles
Theoretical Quantiles

1.3 Radiation

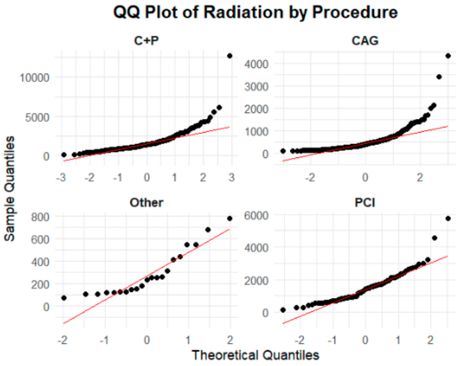

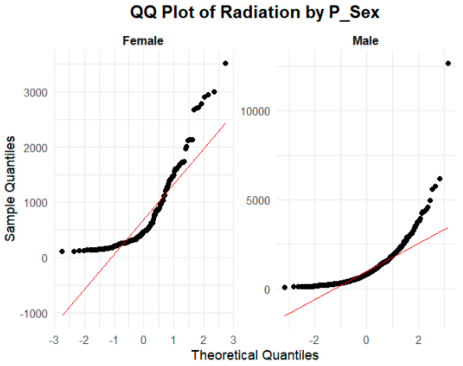

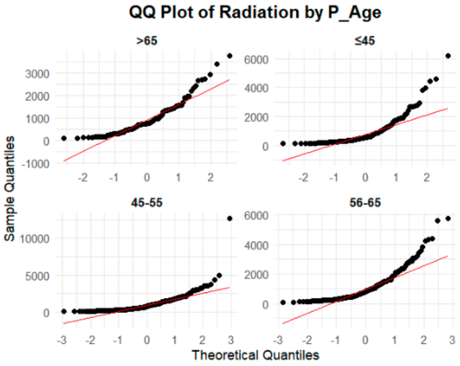

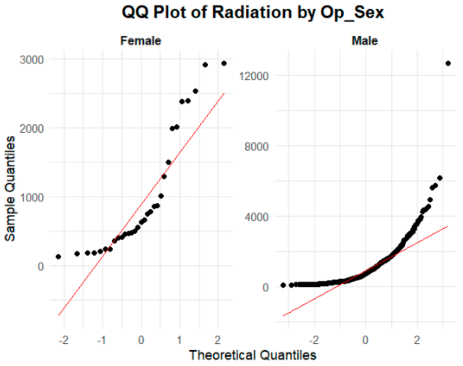

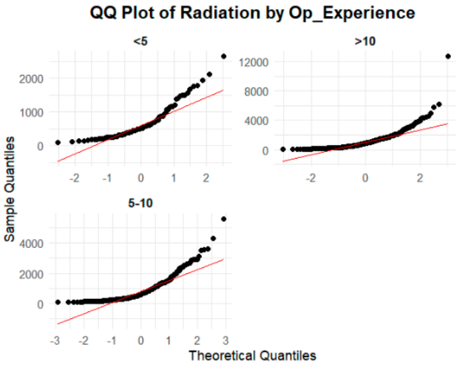

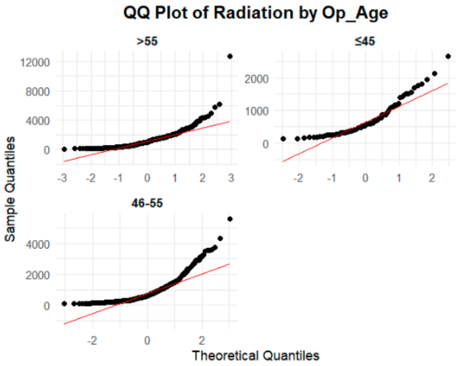

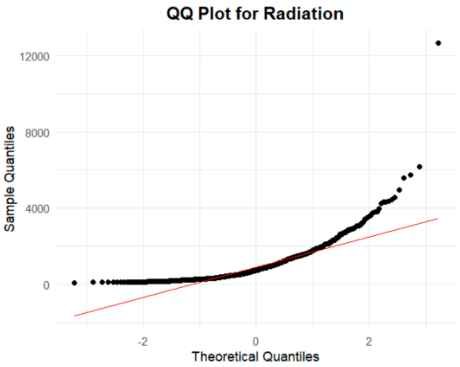

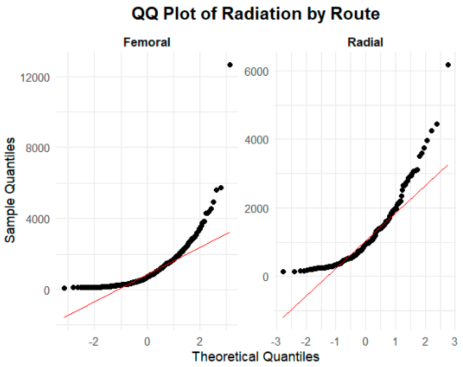

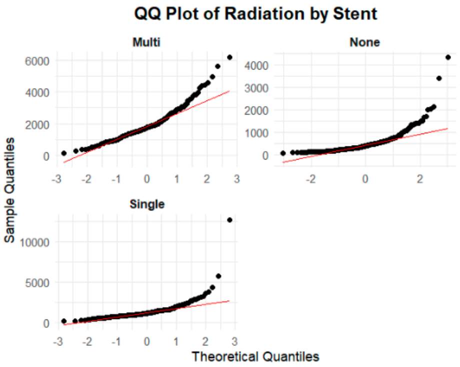

2 Univariate analysis: Kruskal-wallis test with box plots

2.1 View

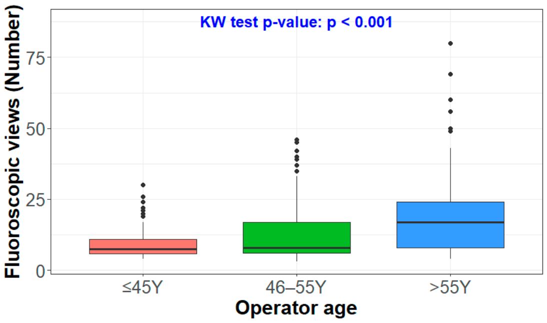

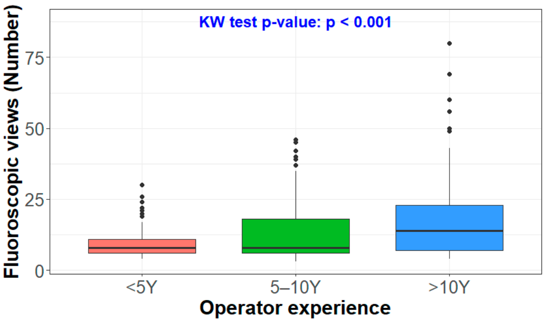

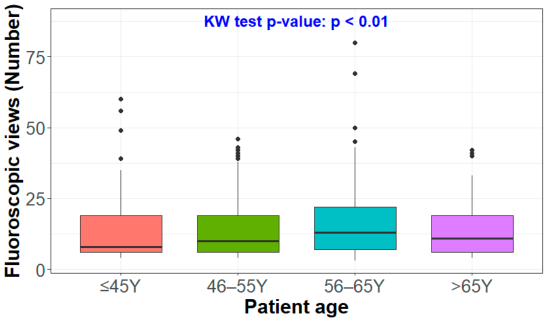

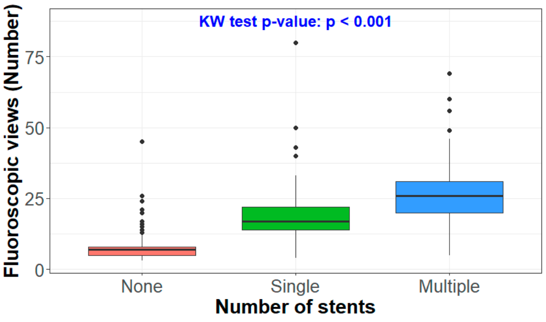

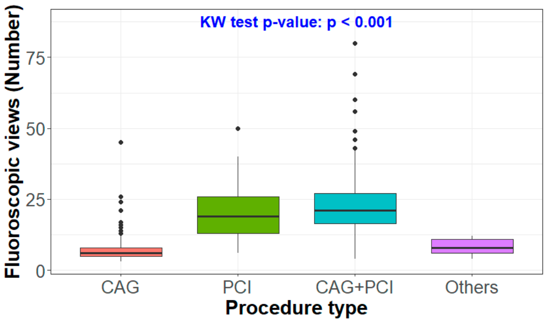

2.2 Time

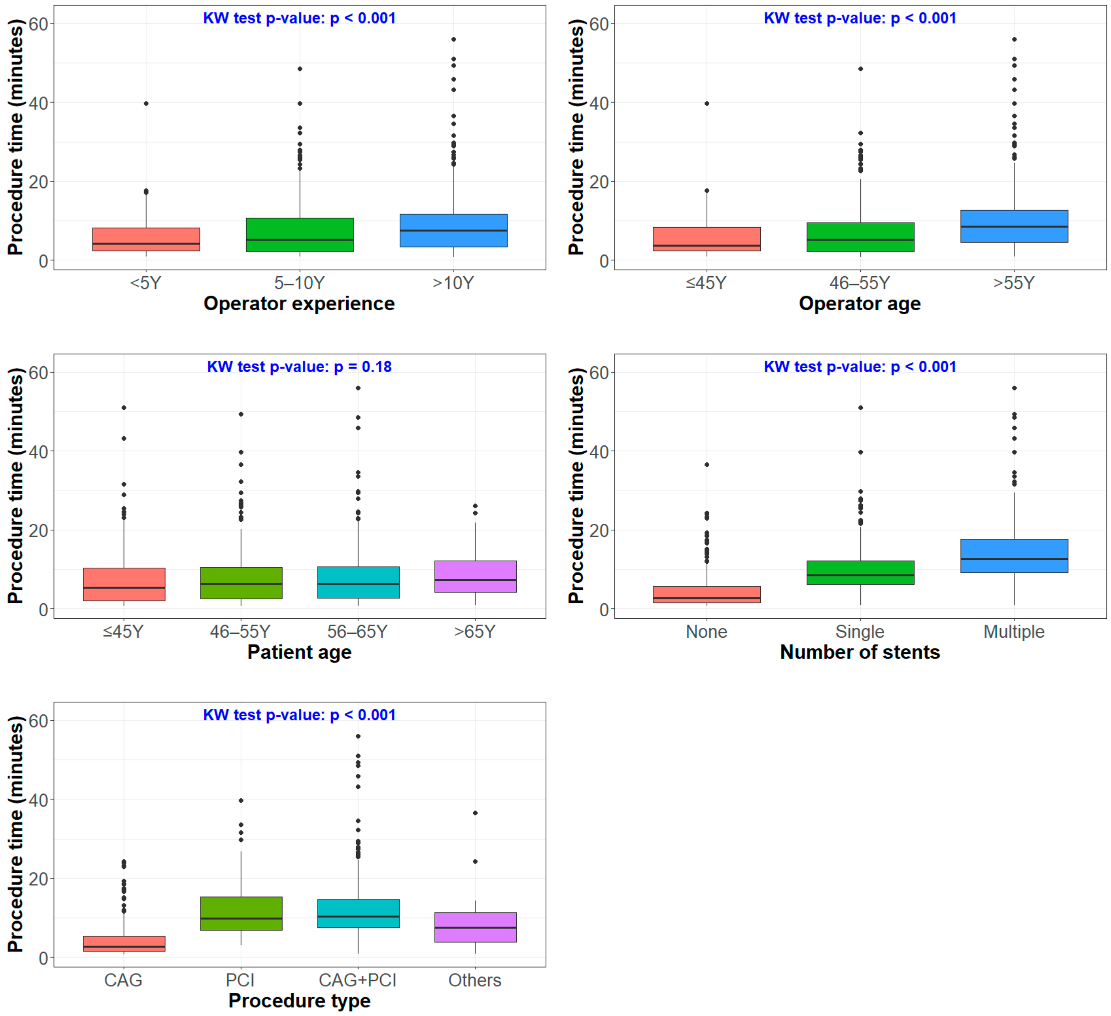
KW test p-value: p < 0.001
Procedure time (minutes)
0
20
40
60
<5Y
5–10Y
>10Y
Operator experience
KW test p-value: p < 0.001
Procedure time (minutes)
≤45Y
46–55Y
>55Y
Operator age
KW test p-value: p = 0.18
Procedure time (minutes)
≤45Y
46–55Y
56–65Y
>65Y
Patient age
KW test p-value: p < 0.001
Procedure time (minutes)
None
Single
Multiple
Number of stents
KW test p-value: p < 0.001
Procedure time (minutes)
CAG
PCI
CAG+PCI
Others
Procedure type

2.3 Radiation

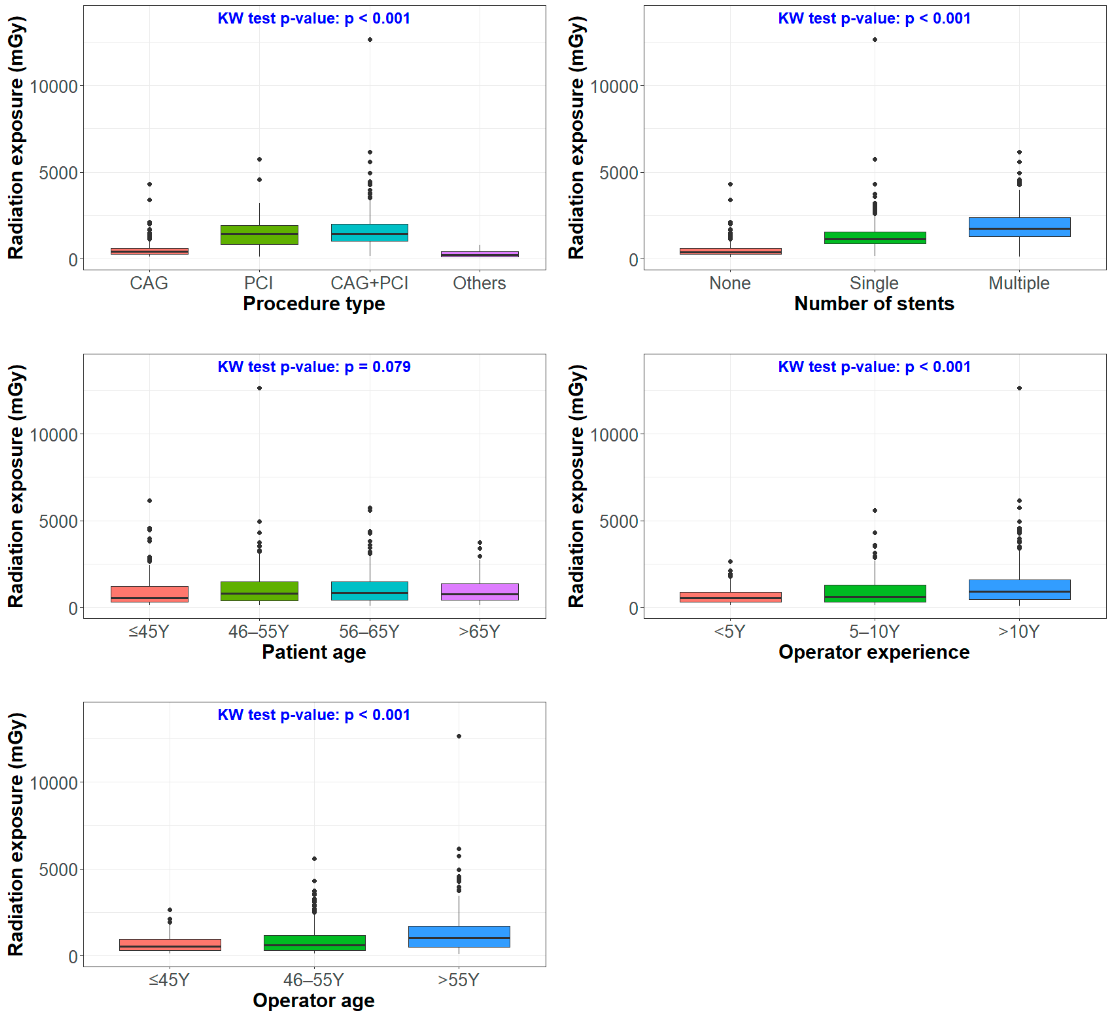

3 Univariate analysis: Wilcoxon rank sum test with box plots

3.1 View

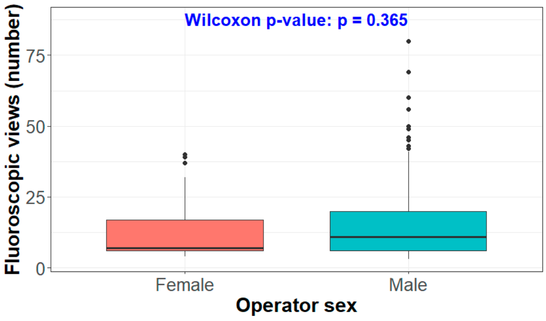

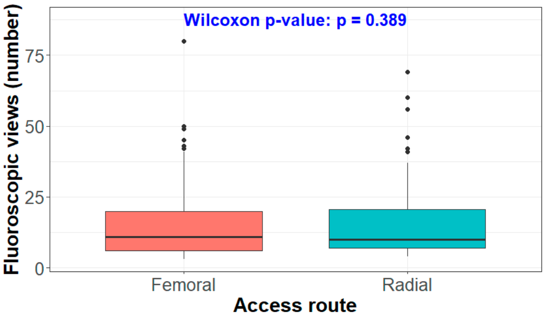

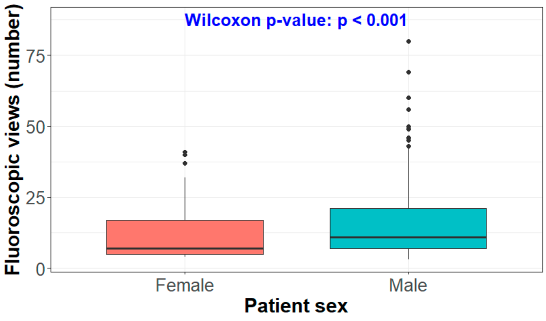

3.2 Time

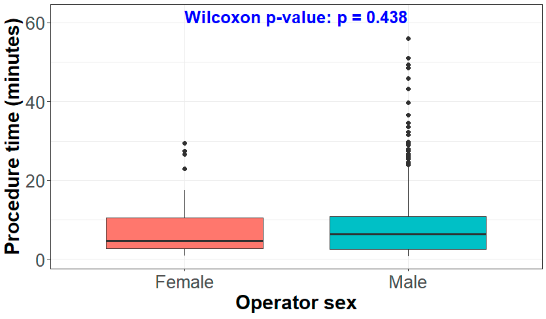

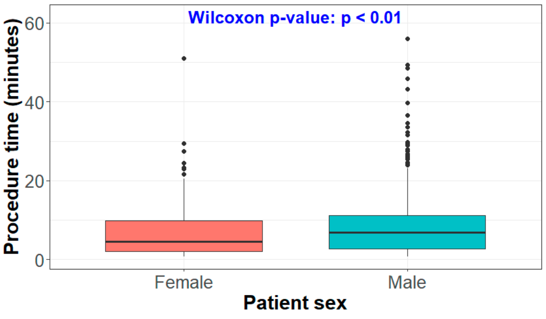

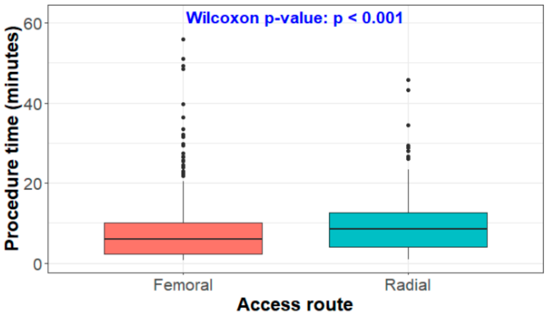

3.3 Radiation

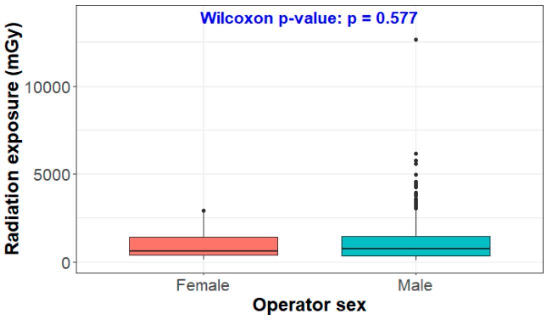

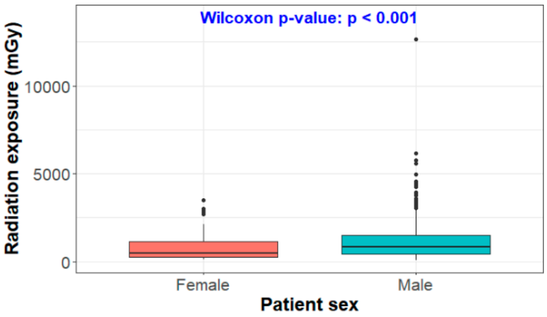

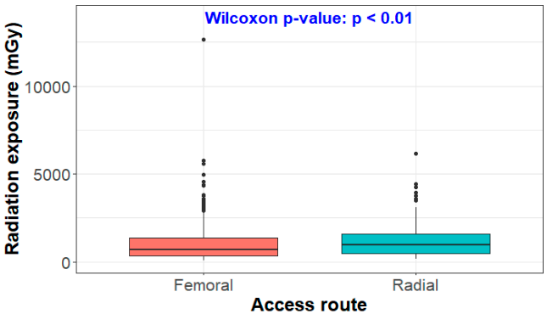

4 Regression model performance and sensitivity analysis

4.1 Residual diagnostics (Residual-versus-fitted)

4.1.1 View

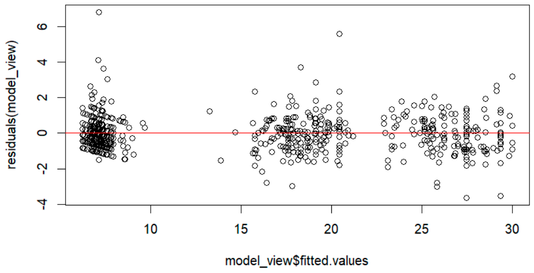

4.1.2 View: better fit

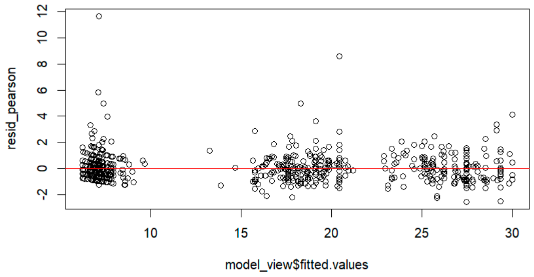

4.1.3 Time

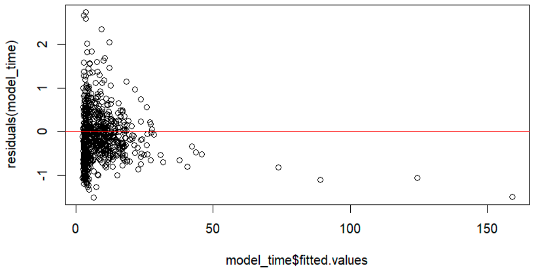

4.1.4 Radiation

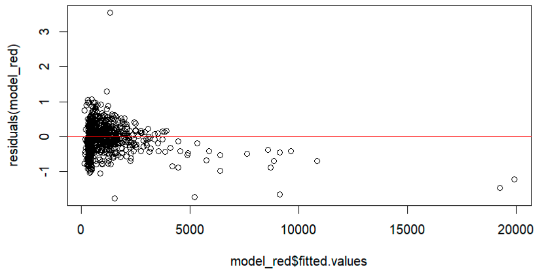

4.2 QQ plots

4.2.1 View

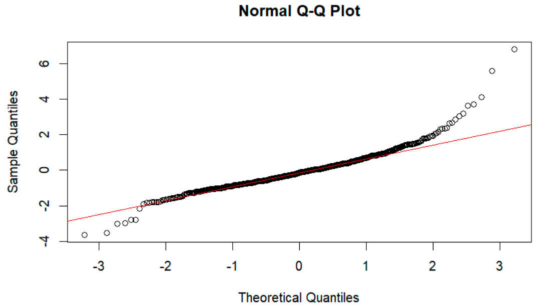

4.2.2 View: better fit

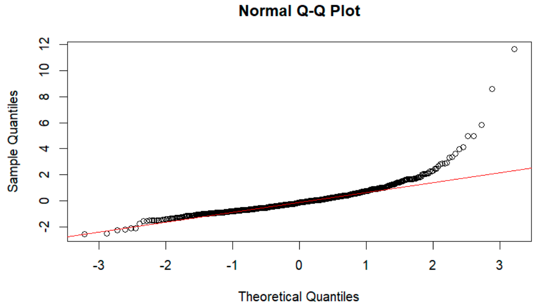

4.2.3 Time

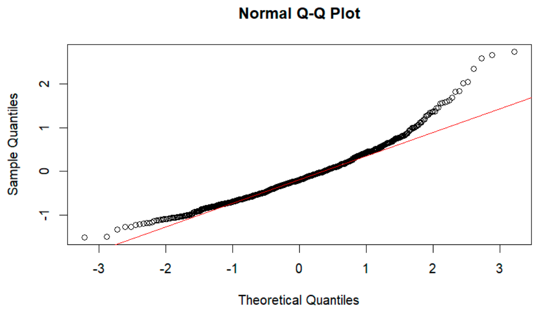

4.2.4 Radiation

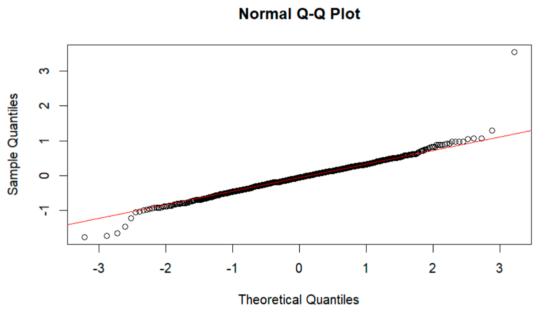

Supplement: Supplementary file 1 [file diagnostics-16-01636-s001.zip › Supplementary figure.pdf]
