# Supplementary material for: Quantifying Radiation Exposure Across Cardiac Catheterization Procedures
Source: Diagnostics (Basel). 2026 May 27;16(11):1636. doi: 10.3390/diagnostics16111636 (PMC13257294; doi:10.3390/diagnostics16111636)
Supplement: Supplementary file 1 [file diagnostics-16-01636-s001.zip › Supplementary tables.pdf]

Supplementary Table S1: Case distribution among the categorial variables

Factors	Variables	Patient Sex		Operator Age (n, %)			Operator Sex (n, %)		Operator Experience (n, %)			Procedure type (n, %)				Accession route (n, %)		Stent (n, %)		
		Female	Male	≤ 45 Y	46 – 55 Y	> 55 Y	Female	Male	< 5 Y	5 – 10 Y	> 10 Y	CAG	PCI	CAG+PCI	Others	Femoral	Radial	None	Single	Multiple
Patient Age	≤ 45 Y	29, 21.0	109, 79.0	15, 10.9	73, 52.9	50, 36.2	4, 2.9	134, 97.1	16, 11.6	56, 40.6	66, 47.8	84, 60.9	19, 13.8	33, 23.9	2, 1.4	99, 71.7	39, 28.3	87, 63.0	25, 18.1	26, 18.8
	46 – 55 Y	85, 27.4	225, 72.6	40, 12.9	152, 49.0	118, 38.1	15, 4.8	295, 95.2	41, 13.2	108, 34.8	161, 41.9	154, 49.7	28, 9.0	119, 38.4	9, 2.9	243, 78.4	67, 21.6	164, 52.9	76, 24.5	70, 22.6
	56 Y – 65 Y	29, 13.0	194, 87.0	18, 8.1	102, 45.7	103, 46.2	7, 3.1	216, 65.9	20, 9.0	72, 32.3	131, 58.2	97, 43.5	31, 13.9	91, 40.8	4. 1.8	178, 79.8	45, 20.2	101, 45.3	66, 29.6	56, 25.1
	> 65 Y	21, 21.0	109, 79.0	3, 2.9	64, 61.0	38, 3.62	5, 4.8	100, 95.2	5, 4.8	38, 36.2	62, 59.0	52, 49.5	11, 10.5	36, 34.3	6, 5.7	77, 73.3	38, 26.7	58, 55.2	30, 28.6	17, 16.2
	p-value	0.000		0.014			0.661		0.107			0.011				0.233		0.036		
Patient Sex	Female			17, 10.4	79, 48.2	68, 41.5	9, 5.5	155, 94.5	17, 10.4	60, 36.6	87, 53.0	105, 64.0	10, 6.1	46, 28.0	3, 1.8	120, 73.2	44, 26.8	108, 65.9	31, 18.9	25, 15.2
	Male			59, 9.6	312, 51.0	241, 39.4	22, 3.6	590, 96.4	65, 10.6	214, 35.0	333, 54.4	282, 46.1	79, 12.9	233, 38.1	18, 2.9	477, 77.9	135, 22.1	302, 49.3	166, 27.1	144, 23.3
	p-value			0.813			0.382		0.929			0.000				0.237		0.000		
Operator Age	≤ 45 Y						2, 2.6	74, 97.4	76, 100.0	0, 0.0	0, 0.0	63, 16.3	2, 2.2	9, 3.2	2, 9.5	53, 8.9	23, 12.8	65, 15.9	2, 1.0	9, 5.3
	46 – 55 Y						29, 7.4	362, 92.6	6, 1.5	166, 42.5	219, 56.0	229, 59.2	43, 48.2	112, 40.1	7, 33.3	296, 49.6	95, 53.1	237, 57.8	91, 46.2	63, 37.3
	> 55 Y						0, 0.0	3.9, 100.0	0, 0.0	254, 82.2	55, 17.8	95, 24.5	44, 49.4	158, 56.6	12, 57.1	248, 41.5	61, 34.1	108, 26.3	104, 52.8	97, 57.4
	p-value						0.000		0.000			0.000				0.107		0.000		
Operator Sex	Female								2, 6.5	0, 0.0	29, 93.5	18, 4.7	2, 2.2	9, 3.2	2, 9.5	27, 4.5	4, 2.2	20, 4.9	6, 3.0	5, 3.0
	Male								80, 10.7	420, 56.4	245, 32.9	369, 95.3	87, 97.8	270, 96.8	19, 90.5	570, 95.5	175, 97.8	390, 95.1	191, 97.0	164, 97.0
	p-value								0.000			0.355				0.249		0.413		
Operator Experience	< 5 Y											67, 17.3	2, 2.2	9, 3.2	4, 19.0	59, 9.9	23, 12.8	71, 17.3	2, 1.0	9, 5.3
	5 – 10 Y											164, 42.4	27, 30.3	78, 28.0	5, 23.8	200, 33.5	74, 41.3	169, 41.2	59, 29.9	46, 27.2
	> 10 Y											156, 40.3	60, 67.4	192, 68.8	12, 57.1	338, 56.6	82, 45.8	170, 41.5	136, 69.0	114, 47.5
	p-value											0.000				0.038		0.000		
Procedure	CAG															292, 75.5	95, 24.5	387, 100.0	0, 0.0	0, 0.0
	PCI															76, 85.4	13, 14.6	0, 0.0	48, 53.9	41, 46.1
	CAG+PCI															208, 74.6	71, 25.4	2, 0.7	149, 53.4	128, 45.9
	Others															21, 100.0	0, 0.0	21, 100.0	0, 0.0	0, 0.0
	p-value															0.010		0.000		
Route	Femoral																	315, 52.8	159, 26.6	123, 20.6
	Radial																	95, 53.1	38, 21.2	46, 25.7
	p-value																	0.199		

Supplementary Table S2: Univariate assessment of View, Time, and Radiation with patient, operator, and procedural predictors.

Variables	Row Labels	Average of View	P value	Average of Time	P value	Average of Radiation	P value
Patient age	>65	13.33±8.95	<0.01	8.49±5.96	0.18	965.11±748.21	0.079
	≤45	13.09±10.58		7.68±8.06		935.30±992.94	
	45-55	13.89±9.40		8.09±7.26		1049.41±1059.86	
	56-65	16.00±11.52		8.41±8.23		1107.71±982.45	
Patient sex	P_Female	11.68±8.87	<0.01	6.93±7.02	<0.01	772.36±723.34	<0.01
	P_Male	14.97±10.49		8.49±7.64		1104.70±1037.93	
Operator experience	<5	9.52±5.66	<0.01	5.84±5.66	<0.01	686.96±531.06	<0.01
	>10	16.35±11.19		8.91±7.79		1178.02±1117.79	
	5-10	12.53±8.95		7.72±7.47		918.41±832.46	
Operator age	>55	17.83±11.75	<0.01	10.20±8.63	<0.01	1306.88±1208.92	<0.01
	≤45	9.57±5.83		5.90±5.81		716.85±538.47	
	46-55	12.38±8.68		6.99±6.46		880.92±794.36	
Operator sex	Op_Female	13.74±11.63	0.365	8.09±8.33	0.438	980.61±881.30	0.577
	Op_Male	14.30±10.20		8.17±7.50		1036.71±993.53	
Procedure type	C+P	22.71±9.70	<0.01	12.52±8.18	<0.01	1681.36±1144.78	<0.01
	CAG	7.08±3.31		4.11±3.87		502.92±406.04	
	Other	8.57±2.68		8.95±8.34		283.54±206.14	
	PCI	20.49±8.86		11.94±7.74		1495.07±905.57	
Accession route	Femoral	14.12±9.98	<0.01	7.62±7.39	<0.01	988.51±988.56	<0.01
	Radial	14.80±11.11		9.98±7.74		1187.72±976.95	
Number of stents	Multi	26.63±9.42	<0.01	14.92±8.94	<0.01	1932.63±1014.19	<0.01
	None	7.19±3.34		4.37±4.34		495.43±407.31	
	Single	18.43±7.88		10.25±6.54		1385.81±1100.46	
	**Grand Total**	**14.28±10.25**		**8.16±7.53**		**1034.46±988.84**	
